# Productive art engagement in a hybrid format: effects on emotions of older adults during COVID-19 pandemic

**DOI:** 10.3389/fpubh.2024.1257411

**Published:** 2024-01-26

**Authors:** Magali Payne, Kevin Galery, Alexandra Plonka, Justine Lemaire, Alexandre Derreumaux, Roxane Fabre, Aurélie Mouton, Guillaume Sacco, Olivier Guerin, Valeria Manera, Philippe Robert, Olivier Beauchet, Auriane Gros

**Affiliations:** ^1^CoBTeK Lab (Cognition Behavior and Technology), Université Cote d'Azur, Nice, France; ^2^Centre Hospitalier Universitaire de Nice, Service Clinique Gériatrique du Cerveau et du Mouvement, Centre Mémoire Ressources et Recherche, Université Côte d'Azur, Nice, France; ^3^Département d'Orthophonie de Nice, Faculté de Médecine de Nice, Nice, France; ^4^Research Center of the Institut Universitaire en Geriatrie de Montreal, University of Montreal, Montreal, QC, Canada; ^5^Département de Santé Publique, Centre Hospitalier Universitaire de Nice, Université Côte d'Azur, Nice, France; ^6^Fédération Hospitalo-Universitaire INOVPAIN, Centre Hospitalier Universitaire de Nice, Université Côte d'Azur, Nice, France; ^7^Université Côte d'Azur, CNRS UMR7284/INSERM U108, Institute for Research on Cancer and Aging Nice, Nice, France; ^8^Department of Medicine, University of Montreal, Montreal, QC, Canada; ^9^Department of Medicine, Division of Geriatric Medicine, Sir Mortimer B. Davis Jewish General Hospital, Lady Davis Institute for Medical Research, McGill University, Montreal, QC, Canada; ^10^Lee Kong Chian School of Medicine, Nanyang Technological University, Singapore, Singapore

**Keywords:** quality of life, wellbeing, apathy, productive art-activity, hybrid format

## Abstract

**Introduction:**

Previous studies have shown benefits of productive art-activity on frail older adults' mental and physical health. In this study, we investigated the effects of art-producing activities in a hybrid format (in-person and online) in a context of lockdown compared with previous studies taking place in museums and their effects on wellbeing, quality of life, physical frailty, and apathy in older adults.

**Methods:**

We conducted a randomized unicentric control trial on a sample of 126 seniors older than 65 years (mean age 71.9 ± 2.3, 81% women) living in Nice (France). Participants were randomized in two parallel groups (intervention group with *n* = 62 vs. control group with *n* = 64) conducted during pandemic, between March and May 2021. The intervention group involved participatory art-based activities conducted in a hybrid format, either in-person or online, once a week for 2 h over a 12-week period. No specific intervention was proposed to the control group. The main aim was to evaluate how this hybrid format would impact the wellbeing, quality of life, and physical frailty of participants. The secondary aim was to compare our results with the previous studies conducted by Beauchet et al., and the third aim was to evaluate the impact of the intervention on apathy. Validated scales were implemented in RedCap and administered at baseline (M0) and at the end of the third month (M3).

**Results:**

The intervention group showed significant improvement in their quality of life (*p* = 0.017) and their level of apathy (*p* = 0.016) after intervention. Emotional blunting increased significantly in the control group (*p* = 0.016) while it remained stable in the intervention group. No significant improvement was observed on the frailty, and wellbeing scores remained constant in both groups.

**Conclusion:**

This randomized control trial confirmed emotional effects on seniors practicing an art-based activity in a hybrid format during pandemic on a weekly basis for 3 months.

**Clinical Trial Registration:**

ClinicalTrials.gov, identifier: NCT04570813.

## 1 Introduction

Because life expectancy has raised consequently, the proportion of older adults is growing fast (1). Living longer in good health condition is a challenge. Involvement in art activities is now well known to improve wellbeing (WB) and quality of life, which are both involved in mental health, linking with longevity and acting as protector factors (2). Life evaluation and hedonic states or emotions are taking a great part in personal experiences and are closely related to mental health. The practice of art-based activities has positive effects on health and wellbeing of older adults (3) and can reduce feelings of loneliness and depressive symptoms, as well as enhance socialization (4). Art therapy is recognized as a valid intervention in mental health, and creativity has been incorporated into gerontology and social sciences of aging, making art practice and health in older adults the foreground of research (5). Creative activities helped to regulate emotions, in particular art and music production (6). Art participants are more likely to use the activity that they found the most helpful as a form of avoidance of negative feelings and a way to socialize with others (7). They can access to web-based activities in order to avoid boredom and feelings of isolation (8). According to Thomson et al. (9), the practice of art-based activities, by allowing the acquisition of new skills, reduce social isolation and decrease anxiety. These activities enhance wellness and happiness scores, and improve emotion and motivation. The practice of art therapies online is a therapeutic process that provides pleasure from the activity as well as the interaction with the art-therapist 9 (10). However the WB and the quality of life can be affected by changes in functional ability, independence and activity performance (4).

The COVID-19 pandemic run worldwide and they were over 14 million deaths (11). Total or partial lockdown had been enacted everywhere across the world; in France, a total lockdown was decided from March 2020 to May 2020, and a partial lockdown was decided from December 2020 to February 2021.

Art-engagement during the COVID-19 pandemic have been associated to people's abilities to cope during lockdown. In that pandemic period, stay-at-home orders were experienced because sanitary reasons, an increasing number of people did suffer from emotional distress, anxiety, depression and loneliness. Art-engagement may have played an important role in people's WB, reducing stress level, lowering level of loneliness and helping escape from negative emotions related to pandemic (12). Art activities could prompt psychological, social and behavioral responses associated with management of WB and mental health. The use of online technologies to provide digital arts in online groups helped older people in a period of emergency (3) and many individuals engaged in art due to a lack of other leisure activities (12).

Emotional wellbeing tends to increase with age, and this tendency was reported also during the COVID-19 pandemic (13), despite older adults being more infected. Behavioral age markers are associated with social withdraw and a reduction in activities that could lead to apathy (14). Apathy in healthy older adults, manifested as a loss of motivation, is very common, which is more than emotional or mental disorders (15). It can affect motivation in three domains, behavioral, social, and emotional, and it is prevalent in varying degrees in healthy people (16).

In previous studies, Beauchet et al. showed that producing art engagement at a museum (17) revealed benefits to frail older adults' mental and physical health.

## 2 Aim and hypothesis

Based on the studies previously conducted by Beauchet et al. (18, 19), the initial project was to replicate in Nice the studies carried out in Montreal and Tokyo. These studies were single-blind randomized control study (RCT) based on artistic productive art engagement at museums of older adults (65+) in the intervention group and life as usual in the control group. Studies were conducted for 3 months, and effects on WB, quality of life and frailty were analyzed. Due to the COVID-19 circumstances, we adapted the way to participate in the artistic productive art engagement, proposing a hybrid way of producing art engagement, either in-person or remote, depending on the orders of Health French Authorities (20). We also modified the place of art engagement because MAMAC was closed, and we performed the in-person activities at the Institut Claude Pompidou in Nice.

Moreover, the first aim of our study was to verify if this hybrid format would allow our sample of older adults to benefit from art-productive activity and show effects on WB, quality of life, and frailty, as it did on the studies by Beauchet and colleagues. The second aim of this study was to determine if art engagement production in a hybrid format in a pandemic period could have an impact on older adults' feelings, interrogating apathy via emotions, behavior, and social practice.

## 3 Materials and methods

### 3.1 Design

Data were collected through Research Electronic Data Capture (REDCap), a secure web application for building and managing online surveys and databases, between January and December 2021. All participants were informed about the subject of the research and the hybrid presentation of the study. They all provided their consent to participate. After providing informed consent, participants completed the study questionnaires. Ethical approval was received from the Institutional Ethics board of Quest III (Clinical Trial Number: NCT04570813).

### 3.2 Participants

The recruitment and follow-up of participants (65+) were carried out in Nice between January and December 2021. Participants were recruited according to three primary approaches: the first used the large senior's database of the City of Nice, the second used the print media coverage in the geriatric hospital and the Center Memoire Recherches Ressources (CMRR), and the third used media diffusion in the local newspaper (Nice-Matin). We got initially 185 responses, 59 were excluded based on the criteria of selection, or some never showed up. The selection criteria were to fulfill the inclusion criteria (being 65 years or older, having access to a smartphone or tablet connected to the Internet, speaking and understanding French, being available during the 3-month period, and being affiliated to the French social security); the non-inclusion criteria were not being able to give or sign consent to participate, show a sensory and/or cognitive deterioration identified by the investigator during inclusion, and not being under guardianship. People were excluded from the study if they withdraw their consent.

**Figure 1:**
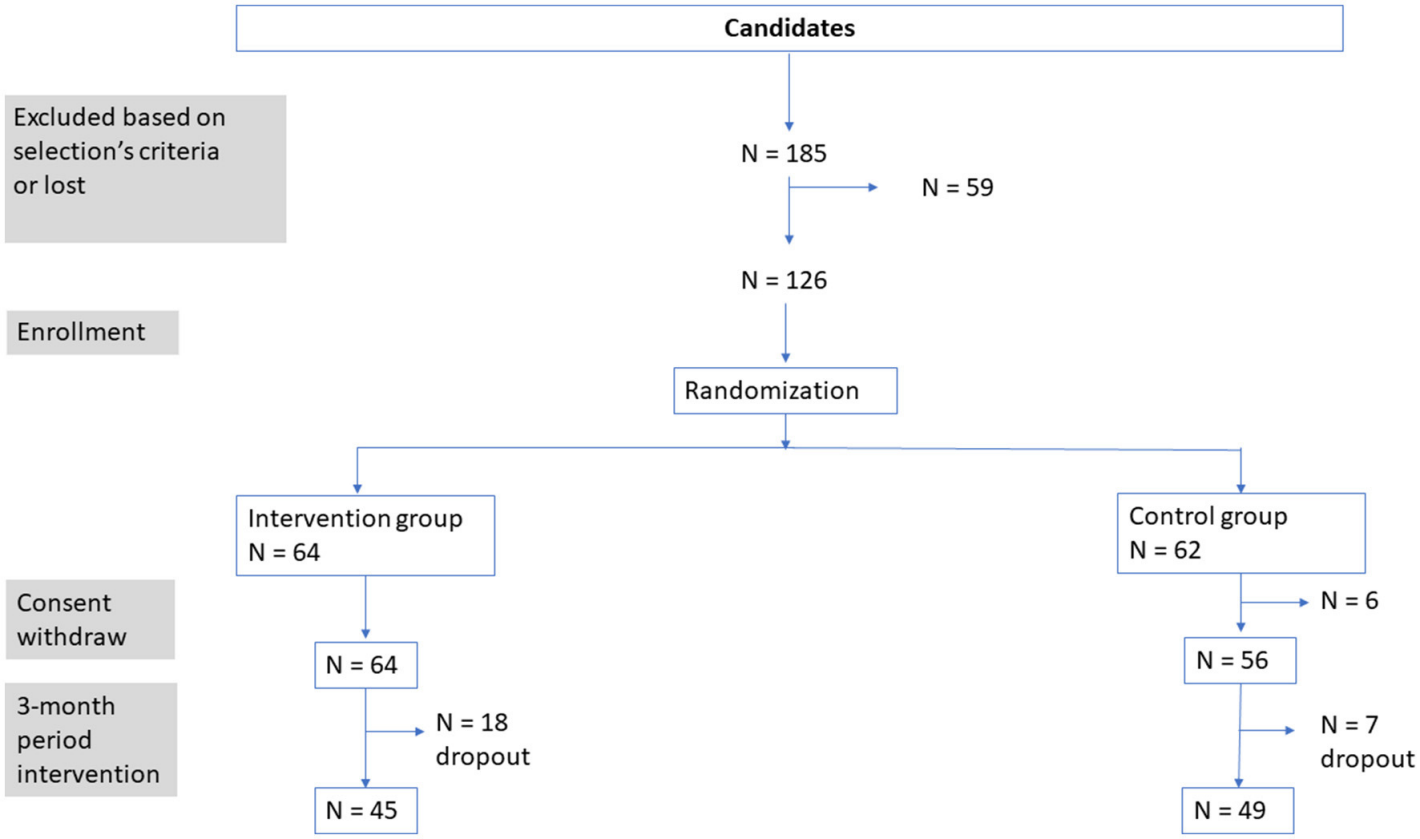
Selection and follow-up of participant's diagram.

A total of 126 people meeting the inclusion criteria were registered to participate in the ART&Santé study by the end of December 2020. The sample size estimation was based on the variation on the wellbeing score before (M0) and after (M3) the study; the difference between the intervention group and control group was estimated to be 5.2 ± 10.3. The minimum number of participants to be able to show this difference between the two groups with a bilateral hypothesis, alpha risk = 5%, and power = 90% is 63 per group. The theoretical number of participants would be 126. Considering an 18% rate of participants lost during the follow-up period, the total number of participants required is 150, but we were not able to reach. The consents were signed between January and February 2021. In total, 64 people were assigned randomly to the intervention group and 62 people were assigned to the control group. In the latter, 6 people dropped out just after the randomization. The inclusion visit had to wait till the end of the local lockdown by the end of February. A total of 126 people underwent the baseline assessment. Overall, 31 participants did not complete final evaluations (*N* = 21) or stopped the protocol (*N* = 10), 13 in the control group and 17 in the intervention group (Figure 1).

### 3.3 Procedure and design

The study was a unicentric (CHU-Nice, Nice, France) RCT, single-blind (principal investigator and representative staff blinded, except the coordinator of the study), randomized, controlled, clinical trial in two parallel groups (intervention group participate in art-based activity vs. control group did not participate in art-based activity but receive a guided tour of the MAMAC at the end of the 3-month period). Participants were randomized into one of the two groups, randomly allocated to the intervention or control group by block randomization with a block size of 1 to limit potential imbalance (Figure 1). The study design was identical to the one described in Beauchet et al. (18), except that the art-activity was provided in-person, or remote, and that the sessions didn't take place at museum, but in the Institute Claude Pompidou. We chose this location because we had enough room to respect Health autorities recommendations. The remote activities were provided via the Internet platform Zoom (Zoom video Communication). The control group participants (no participation to an art-based activity) answered the surveys they received by email link by the end of the third month and by the end of the study they were invited to a guided tour of the MAMAC.

Participants in the intervention group were involved in producing art-based activities in hybrid way 2 h every week for 3 months. Art activities took place in the ICP, with the instructions of half-gauge or online for videoconference. Art activities were always animated by an artist who was chosen by the MAMAC. The public health instructions were constantly adjusted by the Health Authorities.

The intervention consisted of a 3-month cycle of weekly participatory art-based activities such as art-making as part of a group. All participants were engaged in the creative process, allowing them to become authors and observers of others' work. Participants met once a week for 2 h in a dedicated room or videoconference to perform and produce an artistic creation under the supervision of an artist. Sessions took place every morning or afternoon for 1 week, with a maximum of eight participants as request. A total of three artists and three topics were explored, each one during four consecutive workshops. The three topics are music, drawing and painting, and photography and have been organized months before by the MAMAC with artists they were used to work with. We had to provide some adaptations to the initial format because of the remote sessions, especially in the musical sessions.

The musical session (March 2021) was adapted according to the recommendations (20). We offered a free connection to the “SoundTrap” sofware, guided by a written tutorial connection sent by email. People were invited before to connect to Zoom and learnt the way it worked, and then they were individually called by phone when unable to connect to Zoom or SoundTrap. They received three sessions totally remote, and for the last one, they performed in-person group sessions, in accordance with the instructions of half-gauge. They had to produce music, create words, and then sing and perform on percussions together.

The drawing and painting session (April 2021) was partially remote, the instructions for the beginning of the work were given by Zoom on the first session, then participants had to come to the CMRR to get the tools for the second session that was remote, and the two last sessions were in-person groups at ICP, in accordance with the instructions of half-gauge, and people were ask to sculpted flowers and leaves that were picked on the previous sessions and then used as pad to print on fabric.

The photography session (May 2021) was partially remote because sanitary conditions were improving constantly. Only the first session was on Zoom platform; people received instructions and had to take pictures on their own choice. Then, for the last three sessions, they were performing in-person groups at ICP, in accordance with the instructions of half-gauge.

Because some people of this sampler were not in possession of adequate electronic tools or had bad connections or could not use it, the Thursday sessions were totally assisted in the CMRR, in accordance with the instructions of half-gauge, and we provided electronic tablets and in-person assistance to connect to the collective Zoom.

### 3.4 Measures

The baseline assessment (M0) was in-person process using the RedCap program, taking place at ICP before the first workshop, with checking of inclusion criteria and filling in the demographics data (name initials, age, sex, and gender). Then, questionnaires were filled out under the supervision of principal investigator.

The wellbeing was assessed using the Warwick–Edinburg Mental Wellbeing Scale (WEMWBS) self-administered questionnaire (21). This questionnaire is validated and composed of 14 positively worded items with five response categories. It covers most aspects of positive mental health (thoughts and feelings). The score ranges from 14 (none of the time) to 70 (all the time). Cronbach's alpha coefficient was 0.94.

The quality of life was assessed by EuroQol-5D (EQ-5D) (22). This evaluation is a standardized measure of health status that provide a descriptive profile and a single index for health status, composed by a five-item questionnaire in which each question ranging from 1 (no issue) to 5 (worst issue) opening to a summary score between 0 (no issue) and 25 (worst issue), and a visual analog scale represents respondent's self-perceived health ranging from 0 (worth) to 100 (best health). The EQ-5D yield two scores: questionnaire score and VAS score. Cronbach's alpha coefficient was 0.67.

The Center of Excellence Self-Administered (CESAM) questionnaire assessing health and functional status was proposed according to the procedure described in Beauchet et al. (18). This questionnaire summarizes information into two measures: a global frailty score ranging from 0 (absence of deficit) to 18 (all deficits present) and a stratification of frailty in four stages: vigorous (0–3), mild frailty (4–7), moderate frailty (9–13), and severe frailty (>12). Cronbach's alpha coefficient was 0.34.

The Apathy Motivation Index (AMI) (16) was proposed to assess emotions to healthy subjects by indicating the level of apathy and motivation, providing means of different mechanisms underlying sub-clinical lack of motivation in emotional, social, and behavioral domains. This index summarizes three subscales, such as behavioral activation (mean 1.58), social motivation (mean 1.69), and emotional sensitivity (mean 1.05). Cronbach's alpha coefficient was 0.63.

At the end of the workshop sessions (end of the third month, M3), WEMWBS, EQ-5D, CESAM, and AMI were reevaluated online on the RedCap application, each participant was requested via email by sending a connection link.

### 3.5 Statistical analysis

Means, standard deviations (SD), frequencies, and percentages were used to describe participants' characteristics. Inter-group and intra-group comparisons were performed using unpaired and paired *t*-tests, McNemar test, Stuart–Maxwell test, or chi-square tests as appropriate. Changes in questionnaires between M0 and M3 were calculated using the formula: [(score M3) – (score M0)/(score M3+score M0)/2] × 100. Multiple linear regressions were used to examine the association between variations in each questionnaire's scores (used as dependent variables with separated models for each score) and the participatory art-based activity (used as an independent variable) adjusted on participants' baseline characteristics (i.e. age, sex, and group). *P* < 0.05 were considered statistically significant for linear regressions. All statistics were performed using R 4.0.5 software.

## 4 Results

A total of 126 older adults were enrolled in the study, including 64 in the control group and 62 in the art-activity intervention group.

There was no significant difference in baseline between the intervention and control groups in all measures. The characteristics of participants are presented in Table 1.

**Table 1:** Demographic and characteristics of the population at inclusion.

**Demographic and characteristics at inclusion**							
	**Participants** ***N*** = **126**		**Control group** ***n*** = **64**		**Intervention group** ***n*** = **62**		***P value***
	**Mean**	**[SD]**	**Mean**	**[SD]**	**Mean**	**[SD]**	
Age	71.9	[5.3]	71.2	[4.7]	72.8	[5.8]	0.084
Female (%); Male	102	(81.0); (19)	49	(76.6)	53.0	(85.5)	
Wellbeing (WEMWBS) (/70)	54.5	[7.4]	55.0	[6.8]	54.0	[8.0]	0.471
Quality of life (EQ5-D) (/25)	7.6	[2.4]	7.4	[2.0]	7.9	[2.8]	0.277
**Apathy (AMI)**							
Emotions (/4)	2.6	[0.5]	2.6	[0.5]	2.6	[0.5]	0.961
Social (/4)	2.9	[0.6]	2.9	[0.6]	2.9	[0.6]	0.909
Behavior (/4)	3.1	[0.6]	3.1	[0.6]	3.1	[0.6]	0.942
AMI_total (/4)	2.8	[0.3]	2.8	[0.3]	2.8	[0.4]	0.888
Frailty (CESAM) (/18)	2.9	[2.1]	2.6	[2.2]	3.2	[2.0]	0.134
**Frailty Total**							0.398
Vigorous, *n (%)*	79	(65.8)	44	(71.0)	35	[60.3]	
Mildly frail, *n (%)*	38	(31.7)	16	(25.8)	22	[37.9]	
Moderately frail, *n (%)*	3	(2.5)	2	(3.2)	1	[1.7]	

Baseline characteristics of all participants, including age and scores in WEMWBS, EQ-5D, CESAM, AMI, and frailty scores, were recorded.

The results of the control group compared with the intervention group are shown in Table 2. In total, 32 participants did not complete final evaluations by RedCap (*N* = 22) or stopped the protocol (*N* = 10), 15 in the control group and 16 in the intervention group; the dropout rate was finally 25% instead of 18% and reduced the statistical power of the study. All missing data were excluded.

**Table 2:** Comparison of mean wellbeing, quality of life, frailty, and apathy between control and intervention.

**Scores of Wellbeing (WEMWBS), quality of life (EQ-5D), frailty (CESAM) and apathy (AMI) for**												
**control and Intervention groups (mean and** ***p*****-value)**												
	**Participants**										**Control vs. intervention groups** ***p***	
	**Control** ***n*** = **49**					**Intervention** ***n*** = **45**					**M0**	**M3**
	**M0**		**M3**			**M0**		**M3**				
	**Mean**	**[SD]**	**Mean**	**[SD]**	***p***	**Mean**	**[SD]**	**Mean**	**[SD]**	***p***		
Wellbeing (/70)	54.3	[7.4]	54.5	[7.6]	0.411	54.6	[8.6]	55.5	[8.2]	0.681	0.889	0.576
Quality of life EQ-5D(/25)	7.6	[2.1]	6.7	[1.4]	0.004	7.8	[2.4]	6.9	[1.6]	0.017	0.629	0.457
Quality of life: visual analogic scale (/100)	74.1	[13.9]	72.0	[14.8]	0.623	76.8	[17.5]	75.1	[13.8]	0.667	0.416	0.342
Frailty(CESAM) (/18)	2.8	[2.3]	2.4	[2.2]	0.626	3.0	[1.9]	3.0	[2.1]	0.799	0.676	0.266
Vigorous, *n (%)*	28	(57.1)	26	(68.4)	0.717	25	(67.6)	21	(56.8)	0.513	0.837	0.567
Mildly frail, *n (%)*	9	(23.7)	11	(28.9)		11	(29.7)	15	(40.5)			
Moderately frail *n (%)*	1	(2.6)	1	(2.6)		1	(2.7)	1	(2.7)			
**Apathy (AMI)**												
Emotions (/4)	2.6	[0.6]	2.7	[0.5]	0.016	2.5	[0.5]	2.5	[0.5]	0.514	0.540	0.010
Social (/4)	2.8	[0.6]	2.7	[0.5]	0.497	2.9	[0.6]	2.9	[0.6]	0.963	0.426	0.147
Behavior (/4)	3.0	[0.6]	3.0	[0.6]	0.723	3.0	[0.6]	3.0	[0.6]	0.957	0.971	0.754
AMI total (/4)	2.8	[0.3]	2.8	[0.3]	0.556	2.8	[0.3]	2.8	[0.3]	0.733	0.901	0.793

The wellbeing scores (WEMWBS) before and after the workshop neither improve in the control group (*p* = 0.411) nor the intervention group (*p* = 0.681). The quality of life (EQ-5D questionnaire) significantly improved for the control group between M0 and M3 (*p* = 0.004) and the intervention group (*p* = 0.017). There were no differences between the control and intervention groups at M3 (*p* = 0.457). The emotion score of the AMI was significantly different between the control and intervention groups at M3 (*p* = 0.010), with the control group scoring significantly higher (*p* = 0.016) (meaning higher emotional blunting) while the intervention group scores were stable. The frailty scores (CESAM) neither improve after the intervention in the control group (*p* =0.626) nor the intervention group (*p* = 0.799), with a proportion of vigorous participants higher on a non-significant way for the control group (*p* = 0.717) but lower in the intervention group (*p* = 0.513). The proportion of mildly frail was higher at M3 in both groups.

We have performed the analysis of the previous study by Beauchet et al. (18) (Table 3), to analyze the changes between M0 and M3 for the control group vs. intervention group (formula: [(score M3) – (score M0)/(score M3+score M0)/2] × 100). We have found the same results with significant differences only on AMI emotion score (*p* = 0.035) between the two groups.

**Table 3:** Changes in questionnaires between M0 and M3 with the reproduction of the formula by Beauchet et al. (19).

**Evolution of the answers to surveys from M0 to M3 [according to Beauchet et al**. **(****19****)]**							
**Participants**							
			**Control** ***n** =* **49**		**Intervention** ***n** =* **45**		
	**Mean**	**[SD]**	**Mean**	**[SD]**	**Mean**	**[SD]**	***P*** **-value**
Wellbeing (WEMWEBS)	−0.4	[2.7]	0.3	[4.1]	0.3	[4.1]	0.384
**Quality of life (EQ-5D)**							
Questionnaire score (/25)	−2.9	[5.7]	−2.0	[5.5]	−2.0	[5.5]	0.498
Visual analogic scale (/100)	−0.5	[5.6]	−0.1	[6.2]	−0.1	[6.2]	0.777
Frailty (CESAM) (/18)	−3.1	[22.0]	−6.9	[22.6]	0.8	[21.0]	0.131
**Apathy (AMI)**							
Emotions (/4)	0.7	[4.8]	1.8	[4.5]	−0.4	[4.9]	0.035
Social (/4)	−0.2	[4.9]	−0.3	[4.3]	0.0	[5.5]	0.784
Behavior (/4)	0.0	[4.9]	−0.2	[5.2]	0.2	[4.6]	0.710
Total (/4)	0.1	[3.1]	0.3	[3.1]	−0.2	[3.2]	0.516

Finally, multiple linear regressions were used to examine the association between variations in each questionnaire's scores and the participatory art-based activity adjusted on participants' baseline characteristics (e.g., age, sex, and group). We found only a difference in emotion score of AMI with increased emotional apathy in the control group but not in the intervention group (Table 4). A correlation was found between the evolution of the score AMI total and the evolution of the score AMI emotion (Spearman rho=0.56; *p* < 0.001).

**Table 4:** Multiple linear regressions examining the association between variations in each questionnaire's scores.

**Multiple linear regressions examining the**			
**variations in questionnaires' scores**			
	**Intervention**		
	**Adj** β	**[95% CI]**	***p*** **-value**
Wellbeing (WEMWBS)	0.64	[−0.96; 2.24]	0.429
**Quality of life (EQ-5D)**			
Questionnaire score (/25)	1.05	[−1.51; 3.62]	0.415
Visual analogic scale (/100)	−0.15	[−2.89; 2.59]	0.913
Frailty (CESAM) (/18)	6.81	[−3.64; 17.26]	0.198
**Apathy (AMI)**			
Emotions (/4)	−2.45	[−4.56; −0.33]	0.024
Social (/4)	0.24	[−2.02; 2.49]	0.834
Behavior (/4)	0.56	[−1.68; 2.80]	0.620
Total (/4)	−0.48	[−1.91; 0.95]	0.507

## 5 Discussion

The current study examined the effects of 3-month art-based participatory activities on a hybrid way, remotely, or in-person, on wellbeing, quality of life, frailty, and emotions.

The first aim of our study was to determine if art engagement production in a hybrid format in the pandemic period could have an impact on WB, quality of life, and frailty and to compare this impact with the previous studies.

The WB did not improve in our study in any group (intervention or control) and that is consistent with the previous studies (18). The quality of life improved significantly in both groups, with no significant difference between them. This is not consistent with the previous study, where the results revealed a significant variation in quality of life after the 3-month period for the intervention group (p ≤ 0.001) (18). We did assume that art engagement could have effects on the quality of life as it was notified in some studies even in a pandemic period (12), but we can hypothesize that the same evolution in quality of life from both group in our study is related to the improvement in the sanitary context between March and May 2020. These results can also be explained by the insecurity of the context: continuous changes in sanitary measures and impossibility of predicting the modality of the next workshop beforehand. This uncertain context could have affected the intervention group, reducing the improvement in the quality of life. In previous studies conducted online with creative arts, it has been shown that older adults could be more embodied with emotional experiences, but the studies were qualitatively analyzed through thematic analysis with therapeutic environment (10) which is not the case in our study. Even if we provided an individual help by phone to start with, it has been a complicated process and could have modified the benefit expected by the art engagement activity.

The frailty scores were not significantly different in both groups before and after art-based engagement, and it was the same in the previous studies, *p* = 0.086 (18). In details, our data revealed a higher proportion of vigorous participants, but on a non-significant way, in the control group and a lower proportion in the intervention group. This improvement in the frailty score could be linked to emotional stimulation via art-engagement. However, these non-significant results should be interpreted with caution. Indeed, the internal consistency of the CESAM scale (used to assess frailty) was very poor in our sample (Cronbach's alpha = 0.34), suggesting that self-reported frailty may not be completely reliable in our study.

The second aim of this study was to monitor the impact of a 3-month art engagement on emotions via apathy. The pandemic context is a source of severe apathy. In our study, the emotional blunting score raised significantly in the control group between the beginning and the end of the study and remained constant in the intervention group. Given the extremely anxious context caused by repeated lockdown and the loss of the usual social, hobbies, and cultural activities among seniors (23), we can presume that the art engagement proposed by the “ART et Santé” program acted as a protective factor. These results are in line with other studies that have shown that art engagement helps to maintain persistent positive affective state (24). Art engagement is a factor of risk-reducing of mental health problem among older adults (3) and improve emotional functioning (25).

In addition, our study showed that the online producing art-activity is a good way to preserve positive emotions. This is in line with the effects of the 3-month cycle of weekly virtual museum tours (26). Virtual museum tours have demonstrated benefits including positive emotion improvement when proposed in a group setting. Engaging active art-participation can modify perceptions and so far, stimulate positive emotions, no matter which modality. Our conclusions are in accordance with the fact that the distant artistic activities could be used to stimulate emotions, motivation, sensorially and goal-directed activity (20). This study highlights that emotional feelings linked to apathy can benefit from an artistic engagement, and that apathetic people could benefit from interventions even if modalities evolve as long as they allow emotional stimulation (26).

This study was able to highlight some novel aspects related to the ability of proposing art-productive engagement online with older adults in a pandemic period, proposing in-person if sanitary conditions make it possible, with positive effects on emotions by reducing negative feelings.

Despite these promising results, several limitations should be noted.

Our sample was very homogenous in sex, being predominantly composed of women from Nice, making it difficult to generalize our results. However, this is consistent with previous studies having the same rate of women participants (8, 18).

By adapting our study to the pandemic context, we faced a rate of dropout more important than expected, reducing the generalization of the findings to broader situations. The study was initially proposed to take place at a museum but ended to be a hybrid art engagement with no museum involved because of sanitary conditions. People were aware of this situation, but they dropped-off the study when the modality changed for the first time from in-person session to online. This highlights their difficulties to accept the changes. Access to web-based programs is a technology challenge for some older adults and could be a barrier to participate and answer to online questionnaires (8), because it needed specific material and was time-consuming. These may have linked to poor acceptability of the study (20). The remote condition may have affected the abilities of older adults to cope with the situation (12) because they are very motivated to engage in artistic activities in groups, with all the social aspects that this underlies. Their motivation to participate in remote art-activities may have been low, even when remote access was not a problem (7). The way that modalities switched according to sanitary conditions may have increased the level of dropout, and we may assume that it would have been more advantageous to stick on one modality within the same intervention. The other reason that may explain the dropout rate is the control group not being active. People may have engaged in art-activities because they were desperate to find leisure activities in the pandemic period but not willing to engage in a research study. As a result, the generalization of the effects of this study is limited and needs replication.

The level of art-engagement, the acceptability of the study, and the digital abilities of older people were not taken into account, and these are variables that could have influenced the results of this study.

The inclusion of music as online art engagement may have been counterproductive as it is showed as not being the most effective for people aged +65 years (6).

The frailty assessment using the CESAM scale showed very poor internal consistency, suggesting the importance of further studies to properly assess the effect of art engagement on frailty.

The RCT design, the reproduction of the design of previous studies to compare the results, and the fact to successfully carry out a study in the context of COVID-19 pandemic are the main strengths of our study. Nevertheless, these limitations are also a real advantage: they reflect the reality of the possibilities of intervention and assessments during a pandemic context of lockdown.

## 6 Conclusion

We conclude that a hybrid art engagement activity with older adults is a way to enhance emotional stimulation and reduce the risk of apathy in participants in the very special context of pandemic and stay-at-home orders. We also conclude that art-activity productive engagement in a hybrid way could represent an emotional protection factor in the context of a pandemic for older adults and could be predictors of mental health.

Future perspectives would be to reproduce this study out of pandemic context. It would also be interesting to get an active control group. A longer inclusion period would also allow to monitore long-time effects of act-engagement, and increasing the number of participants would provide stronger statistical power to the results.

Health policy should consider helping older adults by using online technologies and to provide productive arts in online groups, even outside the pandemic context, considering that many older adults lived in remote areas and could be beneficial to such sessions.

## Data availability statement

The data analyzed in this study is subject to the following licenses/restrictions: only if demanded to the investigator. Requests to access these datasets should be directed to auriane.gros@univ-cotedazur.fr.

## Author contributions

MP: Conceptualization, Data curation, Methodology, Project administration, Resources, Writing – original draft, Investigation. KG: Conceptualization, Methodology, Project administration, Resources, Writing – review & editing. AP: Data curation, Visualization, Writing – review & editing, Investigation. JL: Project administration, Writing – review & editing, Data curation. AD: Conceptualization, Data curation, Writing – review & editing. RF: Writing – review & editing, Formal analysis, Validation. AM: Writing – review & editing, Supervision. GS: Supervision, Writing – review & editing. OG: Supervision, Writing – review & editing, Funding acquisition. VM: Data curation, Methodology, Writing – review & editing, Investigation. PR: Conceptualization, Methodology, Supervision, Writing – review & editing, Investigation. OB: Conceptualization, Methodology, Resources, Validation, Writing – review & editing. AG: Funding acquisition, Supervision, Writing – review & editing, Conceptualization, Data curation, Investigation, Methodology, Project administration, Resources.

## References

[1] WHO (2015). Available online at: https://iris.who.int/bitstream/handle/10665/186463/9789240694811_eng.pdf?sequence=1

[2] Steptoe A Deaton A Stone AA. Subjective wellbeing, health, and ageing. Lancet. (2015) 385:640–8. 10.1016/S0140-6736(13)61489-0 25468152 PMC4339610

[3] Keisari S Hoffman Y Ring L Palgi Y. The moderating effects of older adults' receptive arts engagement on the association netween resilience and anxiety symptoms during coronavirus breakout. J Nerv Ment Dis. (2021) 209, 443–448. 10.1097/NMD.0000000000001326 34037551 PMC8168708

[4] Colucci E Nadeau S Higgins J Kehayia E Poldma T Saj A. COVID-19 lockdowns' effects on the quality of life, perceived health and well-being of healthy elderly individuals: a longitudinal comparison of pre-lockdown and lockdown states of well-being. Arch Gerontol Geriatr. (2022) 99:104606. 10.1016/j.archger.2021.104606 34896795 PMC8645291

[5] Galassi F Merizzi A D'Amen B Santini S. Creativity and art therapies to promote healthy aging: a scoping review. Front Psychol. (2022) 13:906191. 10.3389/fpsyg.2022.906191 36225688 PMC9549330

[6] Chmiel A Kiernan F Garrido S Lensen S Hickey M Davidson JW. Creativity in lockdown: Understanding how music and the arts supported mental health during the COVID-19 pandemic by age group. Front Psychol. (2022) 13:993259. 10.3389/fpsyg.2022.993259 36275233 PMC9583145

[7] Drake JE Papazian K Grossman E. Gravitating toward the arts during the COVID-19 pandemic. Psychol Aesthet Creat Arts. (2022). 10.1037/aca0000471

[8] Cohen-Mansfield J Muff A Meschiany G Lev-Ari S. Adequacy of web-based activities as a substitute for in-person activities for older persons during the COVID-19 pandemic: survey study. J Med Internet Res. (2021) 23:e25848. 10.2196/25848 33439851 PMC7836908

[9] Thomson LJ Lockyer B Camic PM Chatterjee HJ. Effects of a museum-based social prescription intervention on quantitative measures of psychological wellbeing in older adults. Perspect Public Health. (2018) 138:28–38. 10.1177/1757913917737563 29130869

[10] Keisari S Piol S Elkarif T Mola G Testoni I. *Crafting life stories in* photocollage: An online creative art-based intervention for older-adults. Behav Sci. (2022) 12:1. 10.3390/bs12010001 35049612 PMC8773113

[11] WHO (2023). Available online at: https://www.who.int/publications/m/item/covid-19-epidemiological (accessed December 22, 2023).

[12] Mak HW Fluharty M Fancourt D. Predictors and impact of arts engagement during the COVID-19 pandemic: analyses of data from 19,384 adults in the COVID-19 social study. PsyArXiv. (2020). 10.31234/osf.io/rckp5 33981269 PMC8109242

[13] Bruine de Bruin W. Age differences in COVID-19 risk perceptions and mental health: evidence from a national U.S. survey conducted in March 2020. J Gerontol B Psychol Sci Soc Sci. (2021) 76:e24–9. 32470120 10.1093/geronb/gbaa074 PMC7542924

[14] Chong TT-J. MANERA 2019. Cortex. (2020) 128:326–7. 10.1016/j.cortex.2020.04.001 32370877

[15] Kawagoe T Onoda K Yamaguchi S. Apathy and executive function in healthy elderly—resting state fMRI study. Front Aging Neurosci. (2017) 9:124. 10.3389/fnagi.2017.00124 28536519 PMC5422524

[16] Ang Y-S Lockwood P Apps MAJ Muhammed K Husain M. Distinct subtypes of apathy revealed by the apathy motivation index. PLoS ONE. (2017) 12:e0169938. 10.1371/journal.pone.0169938 28076387 PMC5226790

[17] Beauchet O Cooper-Brown L Hayashi Y Galery K Vilcocq C Bastien T. Effects of “thursdays at the museum” at the montreal museum of fine arts on the mental and physical health of older community dwellers: the art-health randomized clinical trial protocol. Trials. (2020) 21:709. 10.1186/s13063-020-04625-3 32787893 PMC7422616

[18] Beauchet O Cooper-Brown LA Hayashi Y Deveault M Launay CP. Improving the mental and physical health of older community-dwellers with a museum participatory art-based activity: results of a multicentre randomized controlled trial. Aging Clin Exp Res. (2022) 34:1645–54. 10.1007/s40520-022-02139-3 35578103

[19] Beauchet O Bastien T Mittelman M Hayashi Y Hau Yan Ho A. Participatory art-based activity, community-dwelling older adults and changes in health condition: results from a pre–post intervention, single-arm, prospective and longitudinal study. Maturitas. (2020) 134:8–14. 10.1016/j.maturitas.2020.01.006 32143777

[20] Manera V Agüera-Ortiz L Askenazy F Dubois B Corveleyn X Cross L. In-person and remote workshops for people with neurocognitive disorders: recommendations from a delphi panel. Front Aging Neurosci. (2022) 13:747804. 10.3389/fnagi.2021.747804 35126087 PMC8814601

[21] Tennant R Hiller L Fishwick R Platt S Joseph S Weich S. The Warwick-Edinburgh Mental Well-being Scale (WEMWBS): development and UK validation. Health Quality Life Outcomes. (2007) 5:63. 10.1186/1477-7525-5-63 18042300 PMC2222612

[22] Brooks R. EuroQol: the current state of play. Health Policy. (1996) 37:53–72. 10.1016/0168-8510(96)00822-6. 10158943

[23] Dikaios E Sekhon H Allard A Vacaflor B Goodman A Dwyer E. Connecting during COVID-19: a protocol of a volunteer-based telehealth program for supporting older adults' health. Front Psychiatry. (2020) 11:598356. 10.3389/fpsyt.2020.598356 33343425 PMC7738321

[24] Johannsen S. Relationship between art activities and older adult depression. Psi Chi J Psychol Res. (2019) 24:43–51. 10.24839/2325-7342.JN24.1.43

[25] Beauchet O Remondière S Mahé M Repussard F Decavel F Annweiler C. Geriatric inclusive art and length of stay in acute care unit: a case-control pilot study. J Am Geriatr Soc. (2012) 60:1585–7. 10.1111/j.1532-5415.2012.04069.x 22889027

[26] Beauchet O Matskiv J Galery K Goossens L Lafontaine C Sawchuk K. Benefits of a 3-month cycle of weekly virtual museum tours in community dwelling older adults: results of a randomized controlled trial. Front Med. (2022) 9:969122. 10.3389/fmed.2022.969122 36052331 PMC9424501

